# Cytokine and Chemokine Profile in Individuals with Different Degrees of Periportal Fibrosis due to *Schistosoma mansoni* Infection

**DOI:** 10.1155/2012/394981

**Published:** 2012-12-25

**Authors:** Robson Da Paixão De Souza, Luciana Santos Cardoso, Giuseppe Tittoni Varela Lopes, Maria Cecília F. Almeida, Ricardo Riccio Oliveira, Leda Maria Alcântara, Edgar M. Carvalho, Maria Ilma Araujo

**Affiliations:** ^1^Serviço de Imunologia, Complexo Hospitalar Universitário Professor Edgard Santos, Universidade Federal da Bahia, Rua João das Botas s/n, Canela, 40.110-160 Salvador, BA, Brazil; ^2^Departamento de Ciências da Vida, Universidade do Estado da Bahia, 2555 Rua Silveira Martins, Cabula, 41.150-000 Salvador, BA, Brazil; ^3^Instituto Nacional de Ciência e Tecnologia em Doenças Tropicais (INCT-DT-CNPQ/MCT), Rua João das Botas s/n, Canela, 40.110-160 Salvador, BA, Brazil; ^4^Departamento de Análises Clínicas e Toxicológicas, Faculdade de Farmácia, UFBA, Avenida Adhemar de Barros s/n, Ondina, 40.170-970 Salvador, BA, Brazil; ^5^Escola Bahiana de Medicina e Saúde Pública, Rua Frei Henrique No. 08, Nazaré, 40.050-420 Salvador, BA, Brazil

## Abstract

Periportal fibrosis in schistosomiasis has been associated to the host immune response to parasite antigens. We evaluated the immune response in *S. mansoni* infected individuals with different degrees of periportal fibrosis. Cytokine and chemokines were measured in serum and in supernatants of PBMC cultures stimulated with the soluble adult worm (SWAP) or egg (SEA) antigens, using a sandwich ELISA. The levels of IL-5 in response to SEA were higher in individuals with moderate to severe fibrosis (310.9 pg/mL) compared to individuals without fibrosis (36.8 pg/mL; *P* = 0.0418). There was also a higher production of TNF-**α** in cultures stimulated with SWAP in patients with insipient fibrosis (1446 pg/mL) compared to those without fibrosis (756.1 pg/mL; *P* = 0.0319). The serum levels of IL-13 and MIP-1**α** were higher in subjects without fibrosis than in those with moderate to severe fibrosis. However a positive association between serum levels of IL-13, TNF-**α**, MIP-1**α**, and RANTES and *S. mansoni* parasite burden was found. From these data we conclude that IL-5 and TNF-**α** may participate in liver pathology in schistosomiasis. The positive association between IL-13, TNF-**α**, MIP-1**α**, and RANTES with parasite burden, however, might predict the development of liver pathology.

## 1. Introduction

Schistosomiasis is a chronic parasitic infection that affects 200 million people in Africa, South America, and Asia and it is estimated that roughly 700 million people in the world live at risk of infection [1, 2]. In Brazil, the only species described is *Schistosoma mansoni*, and it is estimated that seven million people are infected by the parasite and about 25 million are at risk of infection [3]. The liver pathology results from the host immune response to parasite antigens from the eggs that become trapped in the portal venous system, and it is associated to the morbidity and mortality described in schistosomiasis [4]. 

The granuloma formation around *S. mansoni* eggs is complex and represents an interaction between products secreted by the miracidia, which are released from the egg and the host immune response [5]. Consequently, the granulomas formed act as barriers that prevent the dispersion of egg antigens of *S. mansoni*, and the consequent damage to the liver parenchyma. However, when caused by deposition of large numbers of eggs, the inflammatory process may progress to severe fibrosis, which leads to interruption of normal blood flow in the venous system to the sinusoids resulting in portal hypertension, hepatosplenomegaly, and formation of gastric and esophageal varices that can lead to bleeding and even death. This severe form of the disease occurs in five to ten percent of infected subjects living in endemic areas [6–8].

Some studies have evaluated the immune response associated with granuloma formation and development of periportal fibrosis due to schistosomiasis, both in experimental models and *in vitro* models of granuloma or tissue biopsies [9–11]. 

Recently described, the cytokine interleukin-17 (IL-17) is considered as an inflammatory mediator and may be associated to the pathology of several chronic illnesses [12]. IL-17 stimulates the production of IL-6, nitric oxide, and prostaglandin E2 (PGE2) and acts in synergy with other cytokines, such as IL-1, TNF-*α*, and IFN-*γ*. Furthermore, this cytokine promotes the proliferation and recruitment of monocytes and neutrophils to inflammatory sites. Th17 cells may have been involved in the pathogenesis of schistosomiasis in experimental models [4, 13–15].

There are few studies evaluating the serum levels of cytokines and chemokines in schistosomiasis patients with different degrees of periportal fibrosis and the participation of IL-17 in the formation of granuloma and progression to fibrosis in human schistosomiasis remains unclear. Thus, in this study apart from assessment of Th1, Th2, and T regulatory immune response we also evaluate the levels of IL-17 in human schistosomiasis. 

## 2. Materials and Methods

### 2.1. Study Design and the Endemic Area

This study was carried out in an endemic area from schistosomiasis named Água Preta, in the state of Bahia, Brazil. Água Preta is located 280 km south of Salvador, the capital of the State Bahia. It is composed of a residential area in the center of the village and some surrounding farms. A total of approximately 800 people live in the community. They live in poor sanitary conditions and agriculture is the predominant occupation. There is one river in this region that is used for bathing, washing clothes and utensils, and leisure, exposing the residents to high risk of *Schistosoma* infection.

A cross-sectional parasitological surveys using Kato-Katz [16] and sedimentation techniques were conducted in three different stool samples collected on different days. The current degree of exposure to *S. mansoni* infection was assessed by a previously developed questionnaire, which provides four categories of reported level of exposure to infested water: no exposure, low exposure (<1 h/week), medium exposure (1–3 h/week), or high exposure (1–3 h/day) [17–19].

The inclusion criteria for this study were individuals from endemic areas who have at least one positive parasitological exam for *S. mansoni*. From the 537 individuals who agreed to participate in this study 334 were infected with *S. mansoni* (62.5%). The frequency of other helminthic infections was 43.4% for *Trichuris trichiura*, 37.4% for* Ascaris lumbricoides*, 33.7% for Hookworms, and 3.5% for *Strongyloides stercoralis*. From 334 individuals who were infected with *S. mansoni*, 220 agreed to perform abdominal ultrasound, in order to determine the degree of periportal fibrosis. They also agreed to donate blood for the study of the immunological response. For this particular aim, we did not include individuals under five or above 60 years old. We also did not include alcoholic individuals and those with positive serology for HIV, HTLV-1, or hepatitis virus types B and C, all of which are conditions that could interfere with the immunological response.

### 2.2. Ultrasound Examination

Abdominal ultrasound (USG) were performed using the Quantum 2000 Siemens and Elegra Siemens ultrasound with a convex transductor of 3.5–5.0 Mhz. Liver span was measured in the midclavicular line and midline. The liver was also examined for smoothness of surface, echogenicity and posterior attenuation of the sound bean, and portal vein diameter outside the liver midway between its entrance into the portal hepatic and its first bifurcation in the liver. Periportal fibrosis was observed as multiple diffuse echogenic areas. Grading of periportal fibrosis was determined by the mean total thickness of four portal tracts after the first division from the right and left branches of portal vein (PT1) as follow: degree 0, mean thickness <3 mm; degree I, mean thickness 3 to 5 mm; degree II, mean thickness >5 to 7 mm; and degree III mean thickness >7 mm [20–22]. Of the 220 individuals evaluated 62 (28.2%) had some degree of periportal fibrosis as shown in Table 1. The scores of periportal fibrosis were grouped according to the severity, being degree 0 without periportal fibrosis. Incipient periportal fibrosis was considered to individuals with degree I and moderate to severe periportal fibrosis to those with degrees II and III [23]. 

To perform the immune response we included 30 individuals with degree 0 and 25 individuals with degree I, while all individuals with severe forms of the disease characterized by the grade II (*n* = 13) or III (*n* = 04) were included. 

### 2.3. *Schistosoma mansoni* Antigens

The* S. mansoni* antigens used in this study were the soluble extract of the adult worm of *S. mansoni* (SWAP) and the soluble egg antigen (SEA), prepared as previously described [24].

### 2.4. Cell Culture and Cytokine Measurements

Peripheral blood mononuclear cells (PBMCs) were isolated using Ficoll-Hypaque gradient sedimentation and adjusted to a concentration of 3 × 10^6^/mL in RPMI 1640 medium containing 10% normal human serum (AB positive and heat inactivated), 100 U/mL of penicillin, 100 mg/mL of streptomycin, 2 mmol/L of L-glutamine, and 30 mmol/L of HEPES (all from Life Technologies GIBCO, BRL, Gaithersburg, MS). Cells were cultured without any stimulation or stimulated with 10 mg/mL of SWAP, SEA, or Phytohemagglutinin (PHA). Plates were incubated at 37°C in an atmosphere containing 5% CO_2_. Supernatants were collected after 72 hours of incubation and maintained at −20°C for measurement of cytokines by Enzyme-linked immunosorbent assay (ELISA). Levels of IL-5, IL-13, IL-17, IL-10, IFN-*γ*, and TNF-*α* were determined (R&D Systems, Inc, Minneapolis, MN), and results were expressed as pg/mL on the basis of standard curves.

### 2.5. Serum Cytokine and Chemokine Measurement

Levels of the cytokines IL-5, IL-13, IFN-*γ*, TNF-*α*, IL-17, IL-10, TGF-*β* (R&D systems Inc., Minneapolis) and the chemokines MIP-1*α*/CCL3 and RANTES/CCL5 were measured in serum using sandwich ELISA (eBioscience). The results are expressed as pg/mL, based in standard curves.

### 2.6. Statistical Analysis

Statistical analyses were performed using the Statistical Package for the Social Sciences software (version 9.0 for Windows, SPSS). Statistical differences between groups were assessed using the Kruskal-Wallis analysis of variance test. Fisher's exact test was used to compare proportions. To assess the association between the serum levels of cytokines and chemokines and the parasite burden of *S. mansoni* the Linear Regression tests using Graphpad PRISM 3.03 software (La Jolla, CA, USA) were used. All statistical tests were two-tailed and the statistical significance was established at the 95 percent confidence interval and significance was defined to *P* < 0.05. 

The Ethical Committee of Climério de Oliveira Maternity, Federal University of Bahia, approved the present study. Informed consent was obtained from all study participants or their legal guardians (License number 240/2008).

## 3. Results

### 3.1. Features of the Studied Subject

The demographic characteristics, parasite burden, and the ultrasonography evaluation for periportal fibrosis measurement are shown in Table 1. It was observed that the mean age of individuals with incipient and moderate to severe periportal fibrosis was higher than in individuals without fibrosis (*P* < 0.05). 

There was no significant difference in gender distribution among groups. There was also no significant difference in the levels of exposure to the contaminated water, with medium to high contact to the water being found in 60.5%, 55.3%, and 80.0% of individuals without periportal fibrosis, incipient fibrosis, and moderate to severe fibrosis, respectively (*P* > 0.05).

The *S. mansoni* parasite burden of individuals without periportal fibrosis was higher than in patients with incipient fibrosis (*P* < 0.05). There was no statistically significant difference of being coinfected with other helminths in all groups of patients (Table 1). 

### 3.2. Cytokine Responses Induced by *S. mansoni* Antigens

Levels of IL-5, IL-13, IL-17, IFN-*γ*, TNF-*α*, and IL-10 were measured in supernatant of PBMCs cultures unstimulated and stimulated with SWAP and SEA as shown in Table 2. A significantly higher levels of IL-5 in cultures stimulated with SEA was observed in the group of patients with moderate to severe fibrosis (median levels = 310.9 pg/mL) compared to those without fibrosis (median levels = 36.8 pg/mL; *P* = 0.0418).

Levels of TNF-*α* were significantly higher in supernatants from SWAP-stimulated PBMC of subjects with incipient periportal fibrosis (median levels = 1446 pg/mL) than in individuals without fibroses (median levels = 756.1 pg/mL; *P* = 0.0319). Moreover, the levels of TNF-*α* in nonstimulated cultures were higher in individuals with moderate to severe fibrosis (median levels = 488.7 pg/mL) compared to subjects without periportal fibrosis (median levels = 61.9 pg/mL; *P* = 0.0182). 

The levels of IL-13, IL-17, IFN-*γ*, and IL-10 did not differ significantly between individuals of the different groups (Table 2).

### 3.3. Serum Levels of Cytokines

**Figure 1:**
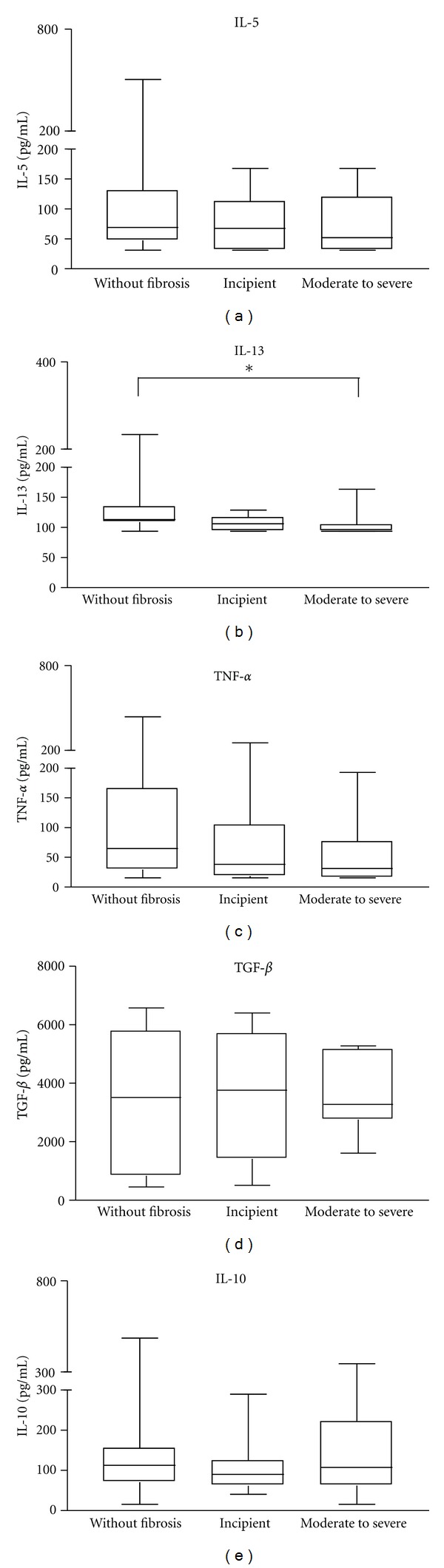
Serum cytokine levels in schistosomiasis individuals with different degrees of periportal fibrosis: (first box) individuals without fibrosis (*n* = 30), (second box) incipient fibrosis (*n* = 25), and (third box) moderate to severe periportal fibrosis (*n* = 17). Levels of IL-5, IL-13, TNF-*α*, TGF-*β*, and IL-10 were determined by sandwich ELISA technique. Horizontal lines represent the median values, boxes represent the 25th to the 75th percentiles, and vertical lines represent the 10th to 90th percentiles. Asterisks indicate statistically significant differences (*P* < 0.05; ANOVA).

The levels of serum cytokines in patients with different degrees of periportal fibrosis are shown in Figure 1. With the exception of IFN-*γ* and IL-17, whose levels were below the detection limit of 15.6 pg/mL and 31.2 pg/mL, respectively, in the three groups of patients (data not shown), high levels of serum cytokines were observed in the different groups of individuals (Figure 1).

While the median levels of IL-13 were higher in individuals without periportal fibrosis compared to those with moderate to severe fibrosis (113.3 pg/mL and 97.7 pg/mL, respectively; *P* = 0.0006), there was no significant difference in the median levels of IL-5, IL-10, TNF-*α* and TGF-*β* between groups (*P* > 0.05; Figure 1). 

### 3.4. Serum Level of the Chemokines 

**Figure 2:**
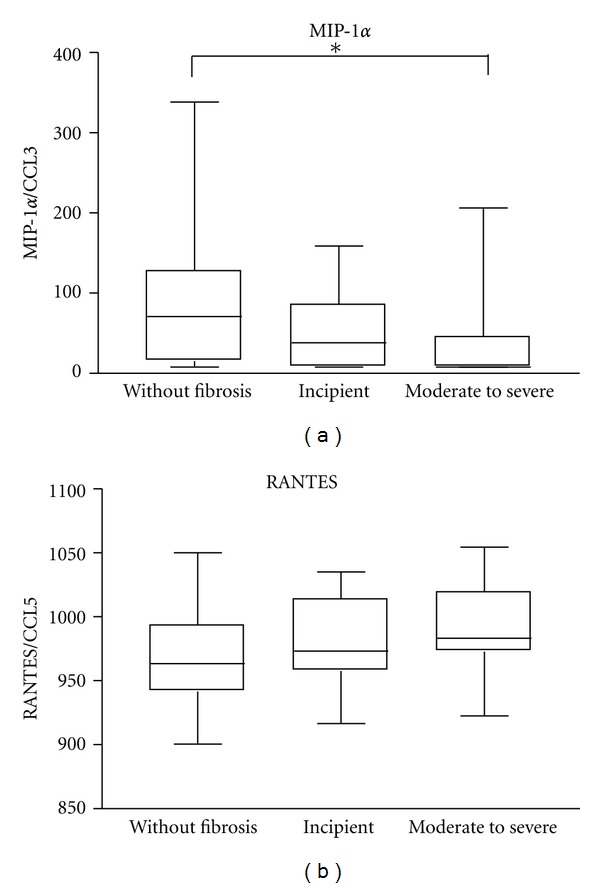
Serum chemokine levels in schistosomiasis individuals with different degrees of periportal fibrosis: (first box) individuals without fibrosis (*n* = 30), (second box) incipient fibrosis (*n* = 25), and (third box) moderate to severe periportal fibrosis (*n* = 17). Levels of MIP-1*α* and RANTES were determined by sandwich ELISA technique. Horizontal lines represent the median values, boxes represent the 25th to the 75th percentiles, and vertical lines represent the 10th to 90th percentiles. Asterisks indicate statistically significant differences (*P* < 0.05; ANOVA).

 The median levels of the chemokines MIP-1*α* and RANTES in serum of individuals with different degrees of periportal fibrosis are shown in Figure 2. It was observed that the median level of MIP-1*α* was higher in individuals without periportal fibrosis (70.9 pg/mL) compared to those with moderate to severe fibrosis (7.8 pg/mL; *P* = 0.0232). There was no significant difference in serum levels of RANTES in individuals with different degrees of periportal fibrosis.

### 3.5. Association between Serum Levels of Cytokines and Chemokines and *S. mansoni* Parasite Burden

**Figure 3:**
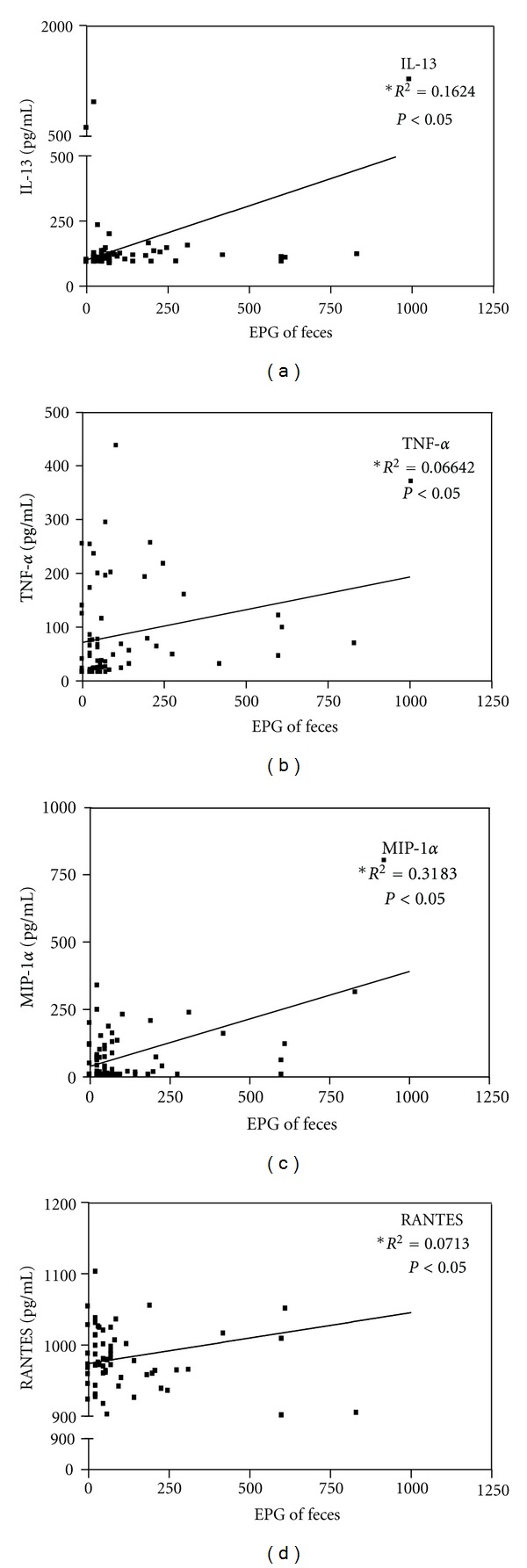
Correlation between serum levels of IL-13, TNF-*α*, MIP-1*α*, and RANTES (*n* = 65) and *S. mansoni* parasite burden. *P* < 0.05 indicates statistically significant differences (Linear Regression).

We tested the possible association between serum levels of cytokines and chemokines and *S. mansoni* parasite burden in the studied population. A positive association between serum levels of IL-13, TNF-*α*, MIP-1*α*, and RANTES and parasite burden was observed (Figure 3). There were no significant associations between serum levels of IL-5 (*R* ^2^ = 0.003413; *P* > 0.05), IFN-*γ* (*R* ^2^ = 0.005831; *P* > 0.05), IL-10 (*R* ^2^ = 0.004651; *P* > 0.05), and TGF-*β* (*R* ^2^ = 0.003391; *P* > 0.05) and the parasite burden of *S. mansoni*.

## 4. Discussion

The development of periportal fibrosis can occur as a result of chronic schistosomiasis infection and accounts for the severe forms of the disease [6]. This fact characterizes schistosomiasis as a serious public health problem. 

The present study aimed to evaluate cytokine and chemokine profile in individuals with different degrees of periportal fibrosis living in the schistosomiasis endemic area of Água Preta, Brazil. Additionally, possible personal and environmental features which could interfere with the development of periportal fibrosis were also evaluated. We showed that despite the high prevalence of *S. mansoni* infection in Água Preta, Bahia, the frequency of severe forms of the disease determined by ultrasonography was low. This could be due to intrinsic characteristics of this population, since there is no report of previous treatment for schistosomiasis in this region. Other possible explanation would be the Cairo's methodology to classify the periportal fibrosis which considers only degrees II and III as severe. We opted to use the Cairo's classification because we have performed previous studies using these parameters and because the physicians who have performed the USG in our studies are very well familiar with this classification.

The majority of individuals who had the most severe degree of periportal fibrosis were over 40 years of age, and there was no influence of gender on periportal fibrosis development. This is in agreement with other authors who have demonstrated that most individuals who develop periportal fibrosis are over 50 years of age [22]. This could be explained by the immune response induced by constant reexposure to the parasite over lifetime, or by the slow process of fibrosis formation. Therefore, younger individuals probably have not been exposed long enough to the cumulative effects of collagen deposition in the periportal tract [25].

Many variables may influence the magnitude of the immune response in human schistosomiasis which includes gender and age [26], intensity of infection [27–30], and genetic characteristics of the population [31]. However, the reason why severe fibrosis develops only in a fraction of the population, which is under the same environmental conditions as others who do not develop fibrosis, remains not well understood. In this study, we found no association between level of exposure to contaminated water and degree of periportal fibrosis. Moreover, individuals without periportal fibrosis had a higher parasite burden than individuals with incipient periportal fibrosis. A possible explanation for this observation is that chronic infection can lead to intestinal fibrosis that impairs the migration of eggs to the intestinal lumen and thereby decreases eggs count in parasitological exams [32].

It has been described that the specific type 2 cytokine pattern is a marker of *S. mansoni* chronic infection in mice and humans, and that this response is involved in the development of periportal fibrosis due to schistosomiasis [21, 33–35]. In this study we evaluated cytokine production by PBMC stimulated with the *S. mansoni* antigens SWAP and SEA as well as its serum levels and correlation with parasite load. We observed a greater variability in cytokines levels, which shows heterogeneity of response to the *S. mansoni *antigens.

Levels of IL-5 in supernatants of *SEA-*stimulated PBMCs were higher in cultures of individuals with moderate to severe periportal fibrosis, when compared to the group without fibrosis. These data are similar to what was observed in a study conducted in schistosomiasis patient's residents in other endemic areas of Bahia [36, 37]. These data suggest that the Th2 immune response is developed and directed to antigens from the eggs. IL-5 has been associated with granuloma formation around *S. mansoni* eggs in the liver, since this cytokine participates in eosinophil growth and activation contributing to a significant source of profibrotic mediators such as IL-13 [38]. Likewise, IL-5 may participate directly in tissue remodeling and fibrosis in schistosomiasis [38]. In contrast to our data that showed no significant difference or correlation between serum levels of IL-5 and *S. mansoni* parasite burden in individuals with different degrees of periportal fibrosis, it has been described that serum levels of IL-5 is higher in patients with severe degrees of periportal fibrosis than in those without fibrosis [39]. 

Many studies in experimental models have shown that IL-13 plays an important role in the development of liver fibrosis. The blockage of IL-13 and IL-4 receptors prevented granuloma development and liver fibrosis in mice [33]. Human studies suggest a correlation between production of high levels of IL-13 and development of more advanced degree of liver fibrosis [36, 37]. However, in this study we did not observe differences in IL-13 levels in supernatants of SWAP or SEA stimulated PBMCs in all groups evaluated. We found, however, higher levels of serum IL-13 in the group of individuals without periportal fibrosis compared to patients with severe fibrosis. It has been suggested that levels of IL-13 tend to be higher during the prefibrotic phase, acting as an inducer of the lesion [35, 39, 40]. The parasite burden is one of many other variables that can influence the magnitude of the immune response in human schistosomiasis [32, 41] and a high parasite burden has been associated with the development of fibrosis [5, 6]. Corroborating with these observation, we also found a positive association between parasite burden and serum levels of IL-13.

There are no published data about the role of IL-17 in development of hepatic fibrosis due to schistosomiasis in humans. As reviewed by Tallima et al. (2009), this cytokine is involved in the development of severe disease by recruitment of neutrophils and macrophages in inflammatory sites, demonstrated only in experimental models [42]. We observed production of IL-17 in cultures stimulated by all the antigens tested without, however, differences among the groups. It is suggested that induction of severe liver disease associated with expression of IL-17 in experimental models, with polarized immune response to a Th1 profile, is dependent on the presence of IL-23, another Th17 cytokine [12, 13]. In this study, we evaluated the IL-17 production in individuals chronically infected by *S. mansoni*, who have a predominantly Th2 immune response, which may explain the low and not detectable levels of IL-17. When evaluating serum levels of this cytokine we also found levels of IL-17 below the detection limit in most individuals, which corroborates the results observed when measuring the supernatants of PBMCs cultures. Moreover, there was no correlation between serum levels of IL-17 and *S. mansoni* parasite load.

Some authors have suggested that IFN-*γ* has certain antifibrotic activities, since this cytokine acts by inhibiting production of extracellular matrix proteins, enhances the activity of collagenase in liver tissue, and downmodulates the Th2 response [10, 43]. In this study, we found no significant differences in levels of IFN-*γ* between groups, and no significant association between parasite burden and serum levels of this cytokine in individuals with different levels of periportal fibrosis. 

Although controversy, TNF-*α* is another cytokine that may participate in the granuloma formation and evolution of fibrotic tissue process. Hoffmann and colleagues (1998) have demonstrated in experimental models that TNF-*α* exerts a protective effect, whereas other authors attribute to the TNF-*α* proinflammatory and profibrogenic effects [7, 44]. In the present study it was observed that cells of individuals with incipient or moderate to severe fibrosis, even without antigenic stimuli, produced higher levels of TNF-*α* when compared to those without fibrosis. Additionally, there was a positive association between serum levels of TNF-*α* and *S. mansoni* parasite burden, which suggests that this cytokine may contribute to the liver pathology observed in schistosomiasis [45]. Supporting the role of TNF-*α* in the development of liver pathology due to schistosomiasis, a study conducted in a schistosomiasis endemic area in Brazil has demonstrated that individuals with moderate to severe periportal fibrosis have higher serum levels of TNF-*α* than those without fibrosis [39]. 

Some studies have pointed out TGF-*β* as an important factor in periportal fibrosis development during chronic schistosomiasis. This cytokine is considered a multifunctional cytokine that regulates biological processes such as inflammation, development, and differentiation of many cell types, tissue repair, and tumor genesis. It is also associated with pro-inflammatory responses and immunosuppressive activities [46, 47] and participates in the process of Th17 cells differentiation [42]. In this study, we found no significant differences in serum levels of TGF-*β* between groups with different degrees of periportal fibrosis, nor did we find an association between parasite burden and levels of TGF-*β*. Recent studies in experimental models have shown a negative association between serum levels of TGF-*β* and *S. mansoni *parasite burden in chronically infected animals, suggesting the involvement of this cytokine in controlling the parasite load [48]. 

Other molecule assessed in this study was the regulatory cytokine IL-10, which is associated with the switch from the Th1 immune response observed during the acute phase to Th2 responses and consequently preventing the development of severe disease. In this study we found no significant difference in the serum levels of this cytokine, nor in the levels measured in supernatant of stimulated cultures among individuals with different degrees of periportal fibrosis. This is in agreement with Silva-Teixeira and coworkers (2004) who demonstrated no association between levels of IL-10 and parasite burden, suggesting that IL-10 may not be involved in periportal fibrosis development during schistosomiasis [39].

Levels of cytokines in patients with schistosomiasis have been evaluated in serum or supernatants of cell cultures. In this study we decided to evaluate cytokine in both, serum and supernatants. We believe that serum cytokine levels represent the *in vivo* profile status. However, as paracrine cytokines, such as IL-5, IL-13, IFN-*γ*, and IL-10, are not well detected in serum using convenient technique such as ELISA, we decided to evaluate these molecules in supernatants of PBMC restimulated *in vitro* with parasite antigens. We believe that these two measurements are complementary and lead to a better knowledge of the cytokine profile in human schistosomiasis.

The role of chemokines in periportal fibrosis due to schistosomiasis is also not completely understood [49]. In this study, we found higher levels of serum MIP-1*α* in individuals without periportal fibrosis than in patients with incipient or moderate to severe fibrosis, which is in contrast to what has been found by Souza and colleagues [50, 51]. We showed, however, a positive association between serum levels of MIP-1*α* and *S. mansoni* parasite burden. This finding is similar to what was obtained for IL-13 and reinforces the thought that individuals without fibrosis enrolled in this study may have been in a prefibrotic stage. Interestingly, it has been also demonstrated that MIP-1*α* induces production of Th2-pattern cytokines, including IL-13 [52, 53]. 

Many authors have suggested that high levels of IFN-*γ* and TNF-*α* are associated with increased expression of RANTES, whereas Th2 cytokines, such as IL-4 and IL-13, are associated with decreased expression [54–56]. In agreement with these studies, it has shown that RANTES deficient mice showed a significant increase granulomatous response when compared to the control group [57]. These studies reinforce the idea that RANTES regulates negatively the development of granuloma and fibrosis. On the other hand, there are no data in the literature on the possible role of RANTES in the development of periportal fibrosis in human schistosomiasis.

The present study showed high levels of IL-5 and TNF-*α* in individuals with periportal fibrosis. Higher levels of IL-13 and MIP-1*α* in individuals without periportal fibrosis were also documented. Considering that these former molecules were positively associated with *S. mansoni* parasite burden, as were TNF-*α* and RANTES, they may represent biomarker for the progression of liver pathology in schistosomiasis. Larger casuistic further studies are needed however to confirm the role of these cytokines and chemokines in the development of periportal fibrosis in human schistosomiasis.

## Figures and Tables

**Table 1:** Demographic characteristics of the individuals enrolled in the study (*n* = 220).

Characteristic	Without fibrosis (*n* = 158)	Incipient fibrosis (*n* = 45)	Moderate to severe fibrosis (*n* = 17)	*P *
Age (median/range)*				
	19 (5–60)	30 (9–58)	40 (21–59)	<0.001^a,b^
Gender *n* (%)**				
Male	83 (52.5)	23 (51.1)	8 (47.1)	ns
Female	75 (47.5)	22 (48.9)	9 (52.9)	
Burden parasite for *Schistosoma mansoni* (EPG/range)*				
	72 (24–4752)	42 (24–1380)	60 (24–200)	0.0451^a^
Coinfection (*Schistosoma mansoni *and other helminth infections) (%)**				
Yes	74.7	68.3	66.7	ns
No	25.3	31.7	33.3	

*Kruskal-Wallis; **Chi-squared Test; ^a^without fibrosis versus incipient fibrosis; ^b^without fibrosis versus moderate to severe fibrosis.

EPG: eggs per gram of feces; ns: not significant (*P* > 0.05).

**Table 2:** Levels of cytokines produced by peripheral blood mononuclear cells (PBMC) of studied individuals.

Cytocines (pg/mL)	Without fibrosis	Incipient fibrosis	Moderate to severe	*P*
median (min–max)	(*n* = 30)	(*n* = 25)	Fibrosis (*n* = 17)	
IL-5				
Without antigen	31.2 (31.2–112.2)	31.2 (31.2–75.5)	31.2 (31.2–127.8)	ns
SWAP	374.4 (31.2–5153.0)	524 (31.2–5905.0)	1988.0 (31.2–5135.0)	ns
SEA	**36.8 (31.2–3820.0)**	188.4 (31.2–4232.0)	**310.9 (31.2–4256.0)**	**0.0418** ^ a^
IL-13				
Without antigen	112.4 (93.8–186.8)	93.8 (93.8–166.0)	93.8 (93.8–93.8)	ns
SWAP	246.6 (93.8–1518.0)	190.3 (93.8 –1290.0)	213.7 (93.8–2091.0)	ns
SEA	171.2 (93.8–1497.0)	93.8 (93.8–1442.0)	93.8 (93.8–1857.0)	ns
IL-17				
Without antigen	15.6 (15.6–132.9)	15.6 (15.6–84.1)	15.6 (15.6–90.7)	ns
SWAP	21.4 (15.6–790.5)	57.1 (15.6–974.8)	21.4 (15.6–345.2)	ns
SEA	18.9 (15.6–291.1)	15.6 (15.6–615.3)	27.2 (15.6–557.5)	ns
IFN-*γ*				
Without antigen	31.2 (31.2–276.2)	31.9 (31.2–1788.0)	31.2 (31.2–777.7)	ns
SWAP	73.1 (31.2–3038.0)	1205.0 (31.2–5507.0)	215.8 (31.2–5249.0)	ns
SEA	83.1 (31.2–288.0)	203.8 (31.2–2905.0)	130.1 (31.2–2714.0)	ns
TNF-*α*				
Without antigen	**61.9 (31.2–633.3)**	346.6 (31.2–2421.0)	**488.7 (31.2**–**3229.0)**	**0.0182** ^ a^
SWAP	**756.1 (80.6–3366.0)**	**1446.0 (236.4–3057.0)**	1002.0 (35.7–2351.0)	**0.0319** ^ b^
SEA	389.8 (102.7–2521.0)	827.3 (31.2–3551.0)	927.3 (89.1–2517.0)	ns
IL-10				
Without antigen	15.6 (15.6–705.5)	48.6 (15.6–1486.0)	17.5 (15.6–1262.0)	ns
SWAP	393.8 (22.1–1976.0)	399.6 (15.6–1779.0)	318.1 (15.6–1213.0)	ns
SEA	585.7 (175.7–2075.0)	1342.0 (15.6–2472.0)	745.8 (35.5–1543.0)	ns

*Kruskal-Wallis; ^a^without fibrosis versus moderate to severe fibrosis; ^b^without fibrosis versus incipient fibrosis; ns: not significant (*P* > 0.05).

SWAP: soluble worm antigen preparation of adult *Schistosoma mansoni*; SEA: *Schistosoma mansoni* egg antigen.
